# Hearing Tests Based on Biologically Calibrated Mobile Devices: Comparison With Pure-Tone Audiometry

**DOI:** 10.2196/mhealth.7800

**Published:** 2018-01-10

**Authors:** Marcin Masalski, Tomasz Grysiński, Tomasz Kręcicki

**Affiliations:** ^1^ Department and Clinic of Otolaryngology, Head and Neck Surgery Faculty of Postgraduate Medical Training Wroclaw Medical University Wrocław Poland; ^2^ Department of Biomedical Engineering Faculty of Fundamental Problems of Technology Wroclaw University of Science and Technology Wrocław Poland

**Keywords:** hearing test, mobile health, mobile apps, pure-tone audiometry

## Abstract

**Background:**

Hearing screening tests based on pure-tone audiometry may be conducted on mobile devices, provided that the devices are specially calibrated for the purpose. Calibration consists of determining the reference sound level and can be performed in relation to the hearing threshold of normal-hearing persons. In the case of devices provided by the manufacturer, together with bundled headphones, the reference sound level can be calculated once for all devices of the same model.

**Objective:**

This study aimed to compare the hearing threshold measured by a mobile device that was calibrated using a model-specific, biologically determined reference sound level with the hearing threshold obtained in pure-tone audiometry.

**Methods:**

Trial participants were recruited offline using face-to-face prompting from among Otolaryngology Clinic patients, who own Android-based mobile devices with bundled headphones. The hearing threshold was obtained on a mobile device by means of an open access app, Hearing Test, with incorporated model-specific reference sound levels. These reference sound levels were previously determined in uncontrolled conditions in relation to the hearing threshold of normal-hearing persons. An audiologist-assisted self-measurement was conducted by the participants in a sound booth, and it involved determining the lowest audible sound generated by the device within the frequency range of 250 Hz to 8 kHz. The results were compared with pure-tone audiometry.

**Results:**

A total of 70 subjects, 34 men and 36 women, aged 18-71 years (mean 36, standard deviation [SD] 11) participated in the trial. The hearing threshold obtained on mobile devices was significantly different from the one determined by pure-tone audiometry with a mean difference of 2.6 dB (95% CI 2.0-3.1) and SD of 8.3 dB (95% CI 7.9-8.7). The number of differences not greater than 10 dB reached 89% (95% CI 88-91), whereas the mean absolute difference was obtained at 6.5 dB (95% CI 6.2-6.9). Sensitivity and specificity for a mobile-based screening method were calculated at 98% (95% CI 93-100.0) and 79% (95% CI 71-87), respectively.

**Conclusions:**

The method of hearing self-test carried out on mobile devices with bundled headphones demonstrates high compatibility with pure-tone audiometry, which confirms its potential application in hearing monitoring, screening tests, or epidemiological examinations on a large scale.

## Introduction

This study investigated the accuracy of the hearing tests conducted on mobile devices calibrated by means of the biological method, that is, in relation to the hearing threshold of a normal-hearing person. This study constitutes the second part of the planned research. The calibration method was adopted from a previous study [1], and it involved semiautomated determination of predefined, model-specific reference sound level.

Hearing loss is a disorder widely encountered among the world population. It is estimated that it affects 5.3% of population, which totals 360 million patients suffering from hearing loss around the world [2]. Monitoring and diagnosis of hearing are especially important in preventing and treatment of hearing loss. One of the basic hearing tests is pure-tone audiometry, which determines the hearing threshold in relation to the sound frequency. However, pure-tone audiometry requires access to specialized medical equipment and staff.

Automated audiometry involves self-determining the hearing threshold and is a valuable diagnostic tool in the case of limited access to medical personnel [3-8]. The methods of automated audiometry have been developed over many years. At present, common methods involve the assessment of the reaction to an automatically generated test signal [4,8-21] or determining the lowest audible sound via self-adjustment of test signal intensity [6,7,9,22]. The hearing thresholds obtained by means of both methods on calibrated devices are comparable with conventional pure-tone audiometry [4-7,9,11-14,19,20,22].

Audiometric tests can be conducted on specialized equipment as well as on generally accessible electronic devices such as personal computers [10,22,23] or mobile devices [4-9,15-21,24,25]. However, reliable results may only be obtained if they are correctly calibrated and conducted in silence [1,15,21,26,27]. Assuming that mobile devices sold with bundled headphones have similar frequency characteristics of the sound system within the devices of the same model, it is possible to share calibration coefficients within these groups. Examinations on iOS-based devices confirmed the validity of automated audiometry based on common calibration coefficients determined in laboratory conditions for all devices of this type [4].

Besides applications typical for automated audiometry such as preliminary evaluation of audiological patients and screening tests [3-8], tests on common electronic devices may be useful for self-monitoring of hearing, especially during recovery from sudden sensorineural hearing loss; in Ménière’s disease, tinnitus or other fluctuating hearing loss, or age-related hearing loss; or during ototoxic therapy [5,6,8,10,16,17,22]. The development of audiometric apps is stimulated by a wide range of potential applications. There is a large number of mobile apps on the market, but only few of them have been investigated in research, which is crucial before they can be clinically applied [28,29].

The objective of this trial was to determine the accuracy of automated audiometry conducted on Android-based mobile sets including the device and the bundled headphones that were calibrated semiautomatically via a biological method. Android-based devices constitute 80% of all mobile devices around the world, and their number is estimated to be 1.4 billion [30]. The prevalence of these devices may contribute to improving accessibility of audiological examinations, especially in the parts of the world with limited access to specialized equipment [5,7,24,31,32]. Android-based mobile devices, contrary to iOS-based ones, are produced by many manufacturers, which means that they are not unified in terms of hardware solutions. This is reflected by statistically significant differences that were found in frequency characteristic of the sound system [1]. Additionally, the number of Android-based models or their variations in 2015 exceeded 24,000, with an increase of 5000 compared with the previous year [33]. Due to the diversity of this group, its size, and dynamics of its substantial changes, laboratory calibration appears to be an inefficient solution.

Calibration of mobile devices conducted by means of a biological method involves determining reference sound level in relation to the hearing threshold of normal-hearing persons [1,34]. Calibration conducted several times on different mobile sets of the same model allows for determining a reliable, model-specific reference sound level [1]. The adopted solution provides support without additional workload not only for the current but also for the future models of mobile sets.

## Methods

This study involved a comparison of hearing thresholds determined by pure-tone audiometry with the hearing thresholds determined on Android-based mobile devices calibrated biologically in uncontrolled conditions. It was a single-center, crossover trial carried out on patients of Otolaryngology Clinic. The consent to conduct the trial has been granted by the Bioethics Committee of Wroclaw Medical University.

### Recruitment

Study participants were recruited offline, using face-to-face prompting. Eligibility assessment was conducted among subjects who owned an Android-based device with bundled headphones. Eligibility criterion, apart from the willingness to participate in the study, included owning the device, for which model-specific calibration coefficient has been previously determined by means of biological method [1]. All the study participants conducted examinations on their mobile devices.

### Measurements

Measurements involved conducting pure-tone audiometry and the test on a mobile device. The order was arranged in a counterbalanced manner. The measurements for both methods were conducted in a sound booth for the frequencies 250 Hz, 500 Hz, 1 kHz, 2 kHz, 4 kHz, 6 kHz, and 8 kHz.

Pure-tone audiometry was conducted by means of conventional 10 dB down and 5 dB up bracketing method (modified Hughson-Westlake method) in accordance with the recommendations of the British Society of Audiology [35]. The measurements were conducted by an audiologist on a clinical audiometer Interacoustic AD229e using TDH-39 headphones (Interacoustics, Denmark, Audiometer Allé, 5500 Middelfart) previously calibrated according to ISO 389-1:1998.

**Figure 1:**
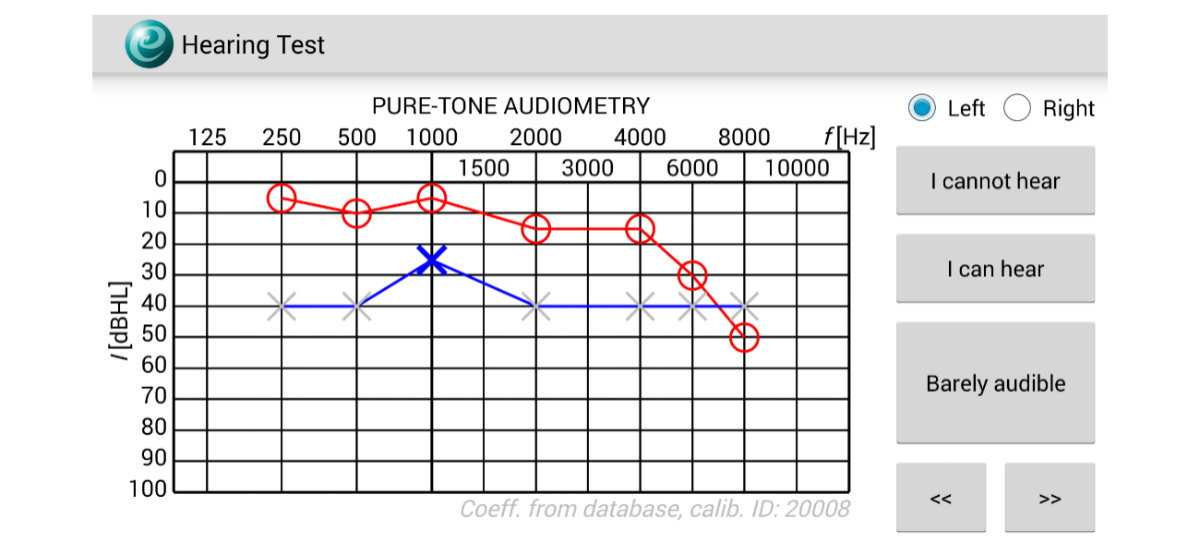
Screenshot of the Hearing Test app during examination.

Hearing examinations on mobile devices were conducted using a free app Hearing Test [36,37] available on Google Play. The examinations involved self-determining of the lowest audible sound generated by the device and were audiologist-assisted. The participant changed intensity of the test sound using the buttons “I can hear” and “I cannot hear” and then confirmed the lowest audible sound using the button “Barely audible.” During the adjustment of the sound intensity, temporary test result was continuously presented on the device screen (Figure 1). The role of the supervising audiologist was to prevent obvious mistakes, such as switching the sides of headphones or omitting the frequency by accidental button pressing. The audiologist also provided assistance in cases of doubts concerning proper execution of the test. Test tone was amplitude modulated with the depth of 100% and the frequency of 2 Hz. Sound intensity was changed in 5 dB steps. When the intensity of the test sound exceeded 40 dB HL, a masking sound was generated contralaterally. The tests on mobile devices were conducted twice: test and retest. Before the retest, the headphones were removed and put on again.

Calibration of mobile devices was described in detail in the previous paper [1]. The objective of calibration was to determine the intensity of the signal generated by the device, which would generate a sound in bundled headphones at the reference level of 0 dB HL. Calibration was performed by means of a biological method, which led to elaboration of a new mobile hearing level (mHL) scale. The level of 0 dB mHL was defined as the sound level generated by the mobile set equivalent to the hearing threshold of normal-hearing persons, reduced by their estimated hearing threshold. The advantage of this approach is the ability to calibrate new device models based on measurements conducted by users themselves, which is essential due to high rotation of mobile models on the market. The calibration is required only for users who are the first to become the owners of new devices, whereas the others can reuse their results.

Calibration measurements involved self-determining the hearing thresholds by means of Bekesy’s method [1,10,11,22,34] by users aged 18-35 years who considered themselves normal-hearing persons. There were no other requirements for calibration. However, if these requirements were not met, a user could ask another person for help. These calibration thresholds were then decreased by the population median to obtain the final reference level. More precisely, the estimator of central tendency of the determined thresholds were approximated by 37th percentile and then decreased by the median of the hearing threshold estimated based on literature data [38] for the corresponding population, that is, aged 18-35 years [1]. Calibration measurements were conducted in uncontrolled conditions, irrespective of this research, before its commencement and when it was in progress, by application users who were not trial participants. To calibrate the model, at least 15 measurements were required to be conducted on various devices belonging to the same model [1]. After each subsequent measurement, the calibration results were automatically updated.

### Statistical Analysis

Sample size was determined on the basis of the standard deviation (SD) between the hearing thresholds in pure-tone audiometry and thresholds determined on mobile devices estimated at 8.42 dB [1]. With the statistical significance of 0.05, statistical power of 0.8, and the effect size of 2.0 dB, the sample volume was estimated on 140 ears (70 subjects). Hearing thresholds obtained on mobile devices have been compared with pure-tone audiometry and analyzed in test-retest examination. Differences in hearing thresholds, intraclass correlation coefficients, and Cronbach alpha with respective confidence intervals have been determined. The results were presented in dependency plots as well as in Bland-Altman plots.

## Results

**Figure 2:**
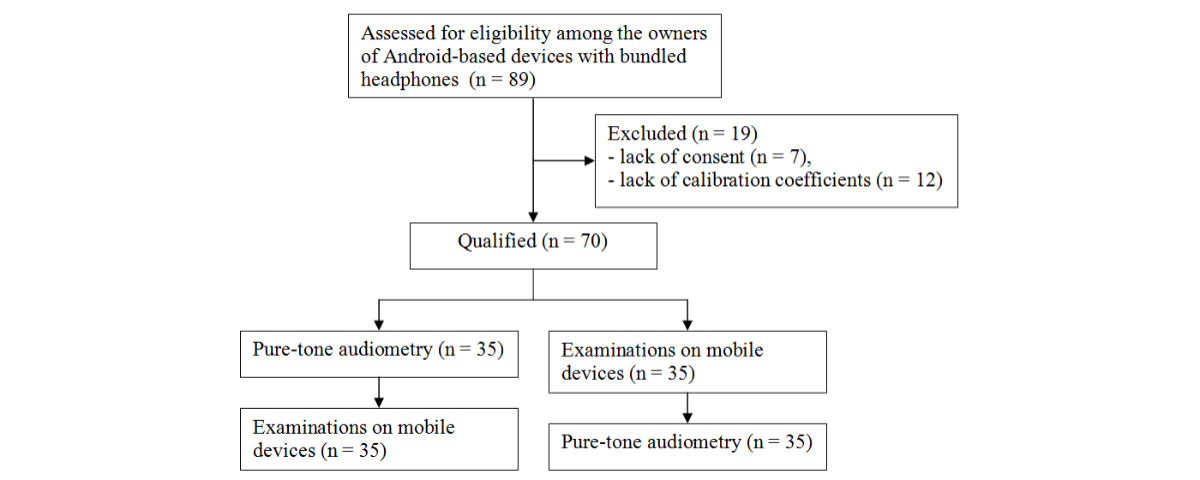
Flow diagram.

In the period between November 11, 2015, and November 2, 2016, 89 subjects were assessed for eligibility from among patients of Otolaryngology Department who owned a mobile device with bundled headphones. Out of 89 subjects, 7 (8%) were not interested in the examination and did not give their consent; 12 out of the remaining 82 subjects (15%) owned devices that were not yet calibrated (Figure 2).

A total of 70 subjects, including 34 men and 36 women, aged 18-71 years (mean 36, SD 11) participated in the trial. Among the participants were both normal-hearing subjects and hearing-impaired patients. Table 1 presents the pure-tone audiometry thresholds for tested frequencies, Table 2 shows distribution of the hearing loss by type, and Table 3 summarizes the models of mobile devices applied.

**Figure 3:**
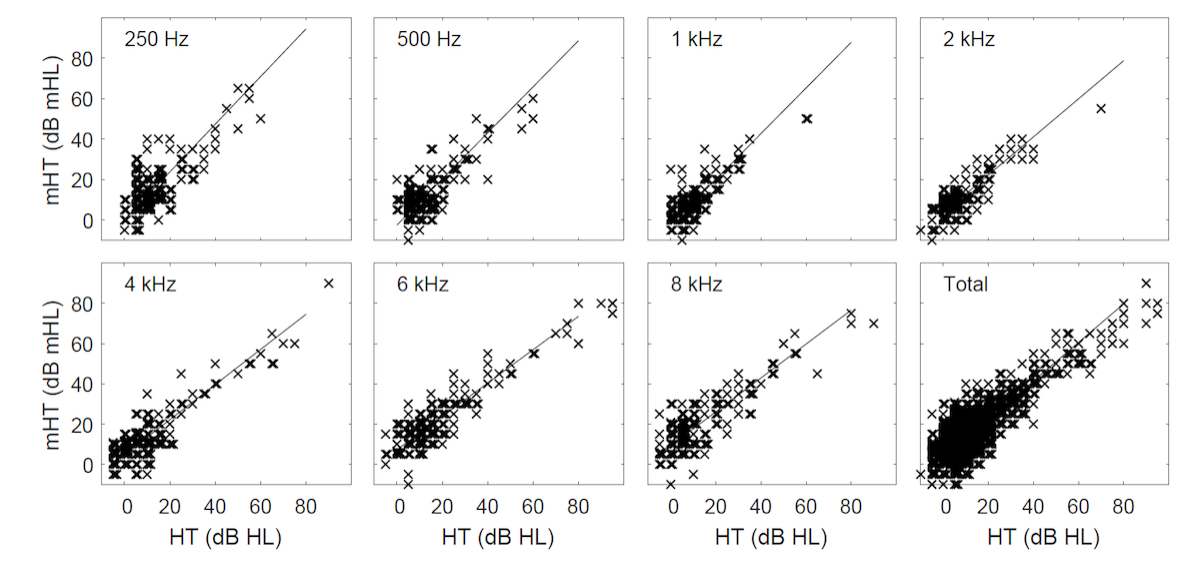
Hearing threshold determined on mobile devices (mHT) in relation to the hearing threshold in pure-tone audiometry (HT).

**Figure 4:**
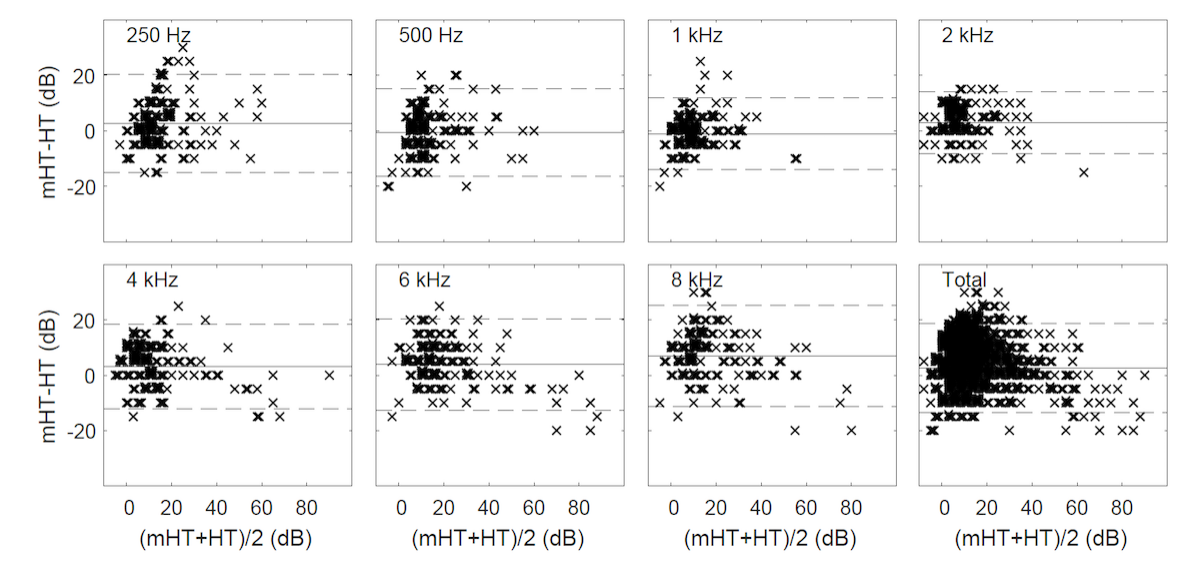
Difference plots (Bland-Altman) between the hearing threshold determined by pure-tone audiometry (HT) and on mobile devices (mHT) (continuous line indicates mean difference; dashed line indicates ±1.96 standard deviation range).

The research was arranged in a counterbalanced manner. Out of 70 subjects, 35 (50%) were first tested by pure-tone audiometry, whereas the other half by the mobile device. Out of the range measurements were discarded from further analysis: 13 of 980 (1.3%), 14 of 966 (1.4%), and 10 of 980 (1.0%) in the case of test, retest, and pure-tone audiometry, respectively. Hearing thresholds determined through pure-tone audiometry were compared with thresholds obtained on mobile devices separately for both groups (Table 4). At the level of statistical significance of *P*=.05, no differences were found between groups, and thus, further analyses were conducted jointly (Tables 5 and 6, and Figures 3 and 4). The mean difference between the hearing threshold determined by pure-tone audiometry and on mobile devices was significantly different from 0 and reached 2.6 dB (95% CI 2.0-3.1), with SD of 8.3 dB (95% CI 7.9-8.7). Intraclass correlation for consistency of single measurement was obtained at the level of 0.85 (95% CI 0.83-0.87), whereas the Cronbach alpha for reliability was calculated at 0.92 (95% CI 0.91-0.93). The number of within-subject differences not greater than 10 dB reached 89% (95% CI 88-91), whereas the mean absolute difference was 6.5 dB (95% CI 6.2-6.9). The largest differences were noted for the frequencies of 6 and 8 kHz at the level of 4.0 dB (95% CI 2.5-5.4) and 7.0 dB (95% CI 5.4-8.6), respectively, whereas the lowest, statistically insignificant at *P*=.05, for the frequency of 500 Hz and 1 kHz.

**Table 1:** Pure-tone audiometry thresholds among study participants.

Hearing loss (dB HL)	Number of ears						
	250 Hz	500 Hz	1 kHz	2 kHz	4 kHz	6 kHz	8 kHz
15 and less	108	108	118	118	110	87	93
16-25	14	16	11	12	12	22	15
26-40	11	11	8	8	7	15	13
41-60	6	4	2	0	4	7	8
60-80	0	0	0	1	5	5	5
Over 80	1	1	1	1	2	4	6

**Table 2:** Type of hearing loss among study participants.

Type of hearing loss		Number of participants
Normal hearing^a^		36
**Sensorineural hearing loss**		
	Sudden deafness	6
	Presbyaccusis	3
	Neuronitis vestibularis	3
	Postviral complication	1
	Other and indefinite sensorineural hearing loss	8
**Conductive and mixed hearing loss**		
	Chronic otitis	9
	Cholesteatoma	2
	Exudative otitis	1
	Other and indefinite conductive or mixed hearing loss	1

^a^Hearing threshold in pure-tone audiometry no higher than 25 dB HL for all tested frequencies.

**Table 3:** Mobile devices by models.

Manufacturer and model	Number of devices
Samsung SM-G900F	7
Huawei ALE-L21	4
Samsung SM-G350	4
Samsung GT-I9300	3
Samsung GT-I9505	3
Samsung SM-A500FU	3
Samsung SM-G386F	3
Samsung SM-G530FZ	3
Samsung GT-I8190N	2
Samsung GT-I9060I	2
Samsung GT-I9515	2
Samsung SM-G800F	2
Samsung SM-A300FU	2
Sony D6603	2
Sony D2303	2
Sony E2303	2
Other	24

**Table 4:** Differences in hearing threshold determined by pure-tone audiometry and on mobile devices depending on the order of examinations.

Frequency in Hz	First pure-tone audiometry		First examination on a mobile device	
	n	Mean difference in dB (95% CI)	n	Mean difference in dB (95% CI)
250	70	3.6 (1.3-5.8)	69	1.8 (−0.2 to 3.8)
500	70	−0.8 (−2.6 to 1.0)	69	−0.4 (−2.5 to 1.6)
1000	70	−0.4 (−2.1 to 1.2)	69	−1.7 (−3.1 to −0.2)
2000	70	2.6 (1.2-3.9)	69	3.3 (1.9-4.7)
4000	70	2.4 (0.4-4.3)	69	4.1 (2.4-5.9)
6000	70	4.0 (2.2-5.8)	69	3.9 (1.6-6.2)
8000	69	6.5 (4.2-8.8)	64	7.5 (5.3-9.7)
Total	489	2.5 (1.8-3.3)	478	2.6 (1.9-3.4)

**Table 5:** Comparison of the hearing threshold determined by pure-tone audiometry and on mobile devices.

Frequency in Hz	n	Mean difference in dB (95% CI)	Standard deviation in dB (95% CI)	Mean absolute difference in dB (95% CI)	Intraclass correlation^a^ (95% CI)
250	139	2.7 (1.2-4.2)	9.0 (8.1-10.2)	6.9 (6.0-7.9)	0.75 (0.67-0.82)
500	139	−0.6 (−2.0 to 0.7)	8.0 (7.2-9.1)	5.9 (5.2-6.8)	0.79 (0.72-0.85)
1000	139	−1.0 (−2.1 to 0.1)	6.6 (5.9-7.4)	4.8 (4.1-5.5)	0.80 (0.73-0.85)
2000	139	2.9 ( 2.0-3.9)	5.7 (5.1-6.4)	4.9 (4.3-5.5)	0.86 (0.82-0.90)
4000	139	3.2 (1.9-4.5)	7.8 (7.0-8.8)	6.7 (5.9-7.6)	0.90 (0.86-0.92)
6000	139	4.0 (2.5-5.4)	8.4 (7.5-9.6)	7.3 (6.4-8.2)	0.90 (0.87-0.93)
8000	133	7.0 (5.4-8.6)	9.3 (8.3-10.6)	9.3 (8.2-10.5)	0.85 (0.80-0.89)
Total	967	2.6 (2.0-3.1)	8.3 (7.9-8.7)	6.5 (6.2-6.9)	0.85 (0.83-0.87)

^a^Two-way random for consistency of single measurement.

**Table 6:** Percentage distribution of within-subject differences in the hearing threshold determined by pure-tone audiometry and on mobile devices.

Frequency (Hz)	n	0-5 dB (%)	0-10 dB (%)	0-15 dB (%)	0-20 dB (%)	0-25 dB (%)	0-30 dB (%)
250	139	74	87	92	96	99	100
500	139	87	94	98	100	100	100
1000	139	93	97	98	99	100	100
2000	139	81	96	100	100	100	100
4000	139	69	91	97	99	100	100
6000	139	66	86	94	99	100	100
8000	133	50	74	89	97	98	100
Total	967	74	89	95	99	100	100

Retest was conducted on a mobile device in 69 of 70 subjects (99%). At the level of statistical significance of *P*=.05, no differences in hearing threshold were found at any frequency (Table 7). The mean difference was −0.1 dB (95% CI −0.4 to 0.2) with SD of 4.4 dB (95% CI 4.2-4.6); 99% (95% CI 99-100) of within-subject differences were within the range of 10 dB, and the mean absolute difference reached 2.8 dB (95% CI 2.6-3.0) (Tables 7 and 8).

The assessment of specificity and sensitivity of hearing loss detection was conducted based on the criterion [39] adopted for the purposes of this study. Hearing loss was diagnosed when the threshold exceeded 30 dB at one of the following frequencies: 500 Hz, 1 kHz, 2 kHz, or 25 dB at more than one, or when the hearing threshold exceeded 50 dB at 4 kHz. The obtained sensitivity was at 98% (95% CI 93-100) with specificity of 79% (95% CI 71-87).

**Table 7:** Comparison of hearing thresholds determined on mobile devices in test-retest examination.

Frequency in Hz	n	Mean difference in dB (95% CI)	Standard deviation in dB (95% CI)	Mean absolute difference in dB (95% CI)	Intraclass correlation^a^ (95% CI)
250	137	0.0 (−0.7 to 0.7)	4.4 (3.9-5.0)	2.9 (2.5-3.4)	0.95 (0.93-0.96)
500	137	−0.3 (−1.0 to 0.5)	4.3 (3.8-4.9)	2.7 (2.3-3.2)	0.94 (0.92-0.96)
1000	137	0.1 (−0.6 to 0.8)	4.3 (3.9-4.9)	2.6 (2.2-3.0)	0.92 (0.89-0.94)
2000	137	−0.4 (−1.0 to 0.2)	3.6 (3.3-4.1)	2.4 (2.0-2.8)	0.94 (0.92-0.96)
4000	137	0.0 (−0.7 to 0.7)	4.2 (3.8-4.8)	2.7 (2.3-3.1)	0.97 (0.95-0.98)
6000	137	−0.1 (−0.9 to 0.7)	4.5 (4.1-5.2)	3.2 (2.8-3.7)	0.97 (0.95-0.98)
8000	130	−0.2 (−1.1 to 0.7)	5.1 (4.6-5.9)	3.2 (2.6-3.7)	0.95 (0.93-0.96)
Total	952	−0.1 (−0.4 to 0.2)	4.4 (4.2-4.6)	2.8 (2.6-3.0)	0.96 (0.95-0.96)

^a^Two-way random for consistency of single measurement.

**Table 8:** Percentage distribution of within-subject differences in the hearing threshold determined on mobile devices in test-retest examination.

Frequency (Hz)	n	0-5 dB (%)	0-10 dB (%)	0-15 dB (%)
250	137	97	99	100
500	137	98	99	100
1000	137	96	99	100
2000	137	99	100	100
4000	137	97	100	100
6000	137	97	100	100
8000	130	97	98	100
Total	952	97	99	100

## Discussion

### Principal Findings

In this paper, hearing thresholds determined by pure-tone audiometry were compared with hearing thresholds determined on Android-based mobile devices previously calibrated biologically in uncontrolled conditions. At the level of statistical significance *P*=.05, no differences in relation to the test order were observed. The mean hearing threshold difference of 2.6 dB (SD 8.3 dB) confirms the reliability of the method. Additionally, the mean test-retest difference of −0.1 dB (SD 4.4) indicates its high repeatability.

### Comparison With Prior Study

The results are consistent with the research presented in previous works. The SD of the difference at 8.3 dB (95% CI 7.9-8.7) corresponds to the value estimated for this calibration method of 8.42 dB [1] and is close to the value of 7.8 dB obtained in another study conducted on the same app Hearing Test [7]. The mean difference of 2.6 dB (95% CI 2.0-3.1) and the mean absolute difference of 6.5 dB (95% CI 6.2-6.9) are also comparable with those presented in [7], that is, 0.7 dB and 7.8 dB, respectively. Contrary to study [7], in which all the tests were carried out on one device, each test in this trial was performed on a different device, thus validating this calibration method.

SD of the difference for the proposed method was 8.3 dB (95% CI 7.9-8.7) with the number of differences no higher than 10 dB at 89% (95% CI 88-91). These values are comparable with the results determined on a set calibrated by a normal-hearing person with verified hearing threshold (SD 6.9 and 8.29 dB [10] and SD 7.88 dB [22]) and are significantly smaller when based on a single biological calibration conducted in uncontrolled conditions (SD 10.66 dB [22]). The highest calibration accuracy is obtained in laboratory conditions, which, however, is not scalable for the Android-based devices. In laboratory conditions, SD varies from 6.4 dB to 9.9 dB [5,6,25], whereas the number of differences not higher than 10 dB changes from 81% up to 96% [4,5,6,19], depending on other settings.

SD of test-retest difference was obtained at 4.4 dB (95% CI 4.2-4.6) with the number of differences no greater than 10 dB at 99% (95% CI 99-100). The results are consistent with other works (SD 4.97 [34], number of differences 97% [6]) using the method of determining the lowest audible sound through self-adjustment of the intensity of the test signal. Moreover, these values do not differ much from the test-retest examinations obtained for automated 10 dB down and 5 dB up bracketing method (SD 6.05 dB and 5.00 dB [34], the number of differences 98% [13]). This confirms the comparable accuracy of both methods [9], at least in the cases of mobile device owners who are familiar with the use of touch screens. Additionally, the results of test-retest examinations confirm the efficiency of the method, especially in self-monitoring of hearing.

**Figure 5:**
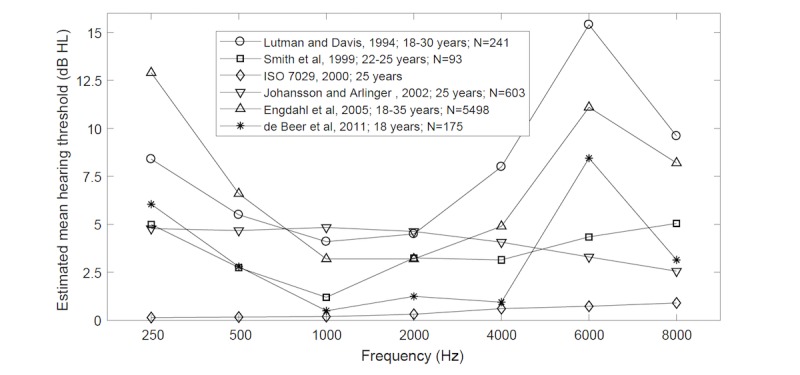
Mean hearing thresholds in a population of young normal-hearing adults estimated on the basis of literature data.

### Limitations

Tests were audiologist-assisted and conducted in a sound booth; therefore, the results are devoid of user mistakes such as omitting the frequency by accidental button pressing or switching the sides of headphones as well as performing the examination in noise. When tested in uncontrolled conditions, care should be taken to minimize the risk of such mistakes, for example, by monitoring the test duration at single frequency and tracking the background noise using a built-in microphone.

The factors limiting the accessibility of the test in relation to the number of Android-based mobile devices are bundled headphones and calibration coefficients. Not all the devices are offered with bundled headphones. Moreover, calibration coefficients for less popular devices may not be determined if the required number of calibrations is not achieved.

During tests on mobile devices, temporary result was continuously displayed on the screen. Therefore, during the retest, subjects could be biased by previous results, consequently reducing the test-retest difference.

Mean difference in the hearing threshold determined through pure-tone audiometry and on mobile devices differed significantly from 0 dB and reached 7.0 dB (95% CI 5.5-8.5) at 8 kHz. The reason for such discrepancies may be related to the literature-based median values of the hearing threshold of normal-hearing subjects, which were adopted to determine 0 dB HL. The mean hearing threshold estimated on the basis of literature data for people aged between 18 and 35 years displays considerable differences (Figure 5) [38,40-44]. It may be related to differing definitions of the normal-hearing person, standards of the measurement method used, or other factors such as genetic composition or occupational distribution of the population. Therefore, the determination of the values for adoption is complex. This research applies values presented in study [38]. In the future, however, it seems rational to correct them by taking into consideration the identified discrepancies (Table 5).

### Conclusions

The method of hearing self-test carried out on mobile devices with bundled headphones calibrated by model-specific coefficients determined in relation to the hearing thresholds of normal-hearing persons demonstrates high compatibility with pure-tone audiometry, which confirms its potential application in hearing monitoring, screening tests, or epidemiological examinations on a large scale. However, one must acknowledge its limitations resulting mainly from the calibration method. Further evaluation of the efficiency of the method, especially in particular applications, is justified and required.

## Abbreviations

HT: hearing threshold determined by pure-tone audiometry
mHL: mobile hearing level
mHT: hearing threshold determined on mobile devices
SD: standard deviation
